# Safety and Effectiveness of an Enhanced Recovery Protocol in Patients Undergoing Burr Hole Evacuation for Chronic Subdural Hematoma

**DOI:** 10.1227/neu.0000000000002849

**Published:** 2024-02-07

**Authors:** Victor E. Staartjes, Antonio Spinello, Nina Schwendinger, Menno R. Germans, Carlo Serra, Luca Regli

**Affiliations:** Machine Intelligence in Clinical Neuroscience (MICN) Laboratory, Department of Neurosurgery, Clinical Neuroscience Center, University Hospital Zurich, University of Zurich, Zurich, Switzerland

**Keywords:** Chronic subdural hematoma, Hygroma, Burr hole, ERAS, Recovery

## Abstract

**BACKGROUND AND OBJECTIVES:**

Enhanced recovery programs may be especially useful in patients with chronic subdural hematoma or hygroma (cSDH), who frequently exhibit frailty and multimorbidity. We aim to evaluate the real-world safety and effectiveness of an enhanced recovery protocol in this population.

**METHODS:**

From a prospective registry, burr hole evacuations for cSDH carried out under the protocol (including early thromboprophylaxis, no flat bed rest, early mobilization without drain clamping, and early resumption of antithrombotic medication) were extracted, along with those procedures carried out within the past year before protocol change. Propensity score–based matching was carried out. A range of clinical and imaging outcomes were analyzed, including modified Rankin Scale as effectiveness and Clavien–Dindo adverse event grading as safety primary end points.

**RESULTS:**

Per group, 91 procedures were analyzed. At discharge, there was no significant difference in the modified Rankin Scale among the standard and enhanced recovery groups (1 [1; 2] vs 1 [1; 3], *P* = .552), or in Clavien–Dindo adverse event grading classifications of adverse events (*P* = .282) or occurrence of any adverse events (15.4% vs 20.9%, *P* = .442). There were no significant differences in time to drain removal (2.00 [2.00; 2.00] vs 2.00 [1.25; 2.00] days, *P* = .058), time from procedure to discharge (4.0 [3.0; 6.0] vs 4.0 [3.0; 6.0] days, *P* = .201), or total hospital length of stay (6.0 [5.0; 9.0] vs 5.0 [4.0; 8.0] days, *P* = .113). All-cause mortality was similar in both groups (8.8% vs 4.4%, *P* = .289), as was discharge disposition (*P* = .192). Other clinical and imaging outcomes were similar too (all *P* > .05).

**CONCLUSION:**

In a matched cohort study comparing perioperative standard of care with a novel enhanced recovery protocol focusing on evidence-based drainage, mobilization, and thromboprophylaxis regimens as well as changes to the standardized reuptake of oral anticoagulants and antiaggregants, no differences in safety or effectiveness were observed after burr hole evacuation of cSDH.

**ABBREVIATIONS:**
cSDH: chronic subdural hematoma or hygroma
CDG: Clavien–Dindo grading
ERAS: enhanced recovery after surgery
KPS: Karnofsky Performance Status
RCT: randomized controlled trial
ITT: intention-to-treat.

Burr hole evacuation of chronic subdural hematoma and hygroma (cSDH) remains one of the most frequent neurosurgical procedures, with increasing incidence among the elderly as demographics shift toward an aging overall population.^1^ Especially in this specific population of patients often burdened with frailty and multimorbidity, minimizing the burden of the surgical procedure and optimizing postoperative recovery is eminently important.

Enhanced recovery after surgery (ERAS) has already swiftly seen wide adoption in the general surgical—especially colorectal—as well as in the vascular surgical specialties, with promising improvements in length of hospital stay, adverse event rates, and clinical outcomes with secondary reductions in healthcare resource utilization and costs.^2-5^ ERAS protocols usually consist of a range of multidisciplinary, evidence-based elements that reach from optimization of a patient's health state up to reducing the physiological burden of the surgery itself and improving the postoperative rehabilitation process. In neurosurgery, ERAS protocols have so far primarily been focused on intracranial tumor and spinal surgery.^5-8^

In October 2022, we implemented a novel institutional protocol (Table 1) implementing several ERAS elements, governing the preoperative, perioperative, and postoperative care of patients undergoing surgery for cSDH. Although some ERAS elements—such as patient blood management—had already been implemented before in our previous, “standard protocol,” the changes to this protocol focus on evidence-based drainage, mobilization, and thromboprophylaxis regimens as well as changes to the standardized reuptake of oral anticoagulants and antiaggregants. Therefore, the aim of this study is to evaluate the safety and effectiveness—thus the real-world impact—of implementing an enhanced recovery protocol for burr hole evacuation of cSDH in a propensity-matched cohort study based on our prospective institutional registry.

**TABLE 1.** Overview of the Standard Protocol and the Subsequently Implemented ERAS Protocol

Time point	Element	Standard protocol	ERAS protocol
Preoperative			
1	Patient blood management	In cases of anemia: for elective procedures according to institutional patient blood management protocol^27^	In cases of anemia: for elective procedures according to institutional patient blood management protocol^27^
2	Laboratory testing: hemostasis	Preoperative testing of thrombocyte count, plasmatic coagulation, and fibrinogen. Substitution if necessary. In case of antiaggregation or anticoagulation, elective cases are put on hold and emergency cases are carried out under reversal	Preoperative testing of thrombocyte count, plasmatic coagulation, and fibrinogen. Substitution if necessary. In case of antiaggregation or anticoagulation, elective cases are put on hold and emergency cases are carried out under reversal
3	Cranial imaging	Max. 2 weeks old must be available	Max. 2 weeks old must be available
4	Prophylaxis against infection	Preoperative cefuroxime or clindamycin	Preoperative cefuroxime or clindamycin
5	Dexamethasone	Not used^26^	Not used^26^
Intraoperative			
6	Prevention of fluid disbalance and blood transfusion	Yes	Yes
7	Prevention of hypothermia	Yes	Yes
8	Extended intraoperative monitoring	Yes	Yes
9	General vs local anesthesia	In most cases, general anesthesia is used. In select cases of multiple comorbidities in adequate patients without aphasia undergoing unilateral surgery, the procedure can be carried out under local anesthesia	In most cases, general anesthesia is used. In select cases of multiple comorbidities in adequate patients without aphasia undergoing unilateral surgery, the procedure can be carried out under local anesthesia
10	Burr hole location	Frontal and parietal	Frontal and parietal
11	Drainage site	Subperiosteal	Subperiosteal. No drainage is left in place if the brain immediately approximates the calvarium intraoperatively^10-12^
12	Burr hole coverage	No recommendation	In patients without hair over the frontal burr hole, use of a burr hole cover can be considered^13^
Postoperative			
13	Postoperative surveillance	For at least 6 hours—but usually 1 night—observation on our neurosurgical intermediate care unit, then transfer to neurosurgical ward	For at least 6 hours—but usually 1 night—observation on our neurosurgical intermediate care unit, then transfer to neurosurgical ward
14	Prophylaxis against thrombosis	48 hours after surgery (after drainage removal) start 5000 I.U./day dalteparin s.c	6 hours postoperatively, start 5000 I.U./day heparin i.v. drip, which is stopped 2 hours before removal of the surgical site drain. On the day of drain removal, 5000 I.U./day dalteparin s.c. is started at 6 pm^17^
15	Surgical site drainage protocol	Attachment of the drainage bag to the bed at neck/shoulder height. Removal after 48 hours in supine position	Drainage bag at heart level. Removal after 36–48 hours in supine position
16	Mobilization	Strict bed rest in flat supine position for 48 hours (if not feasible, max. 20° upper-body elevation), only in select cases earlier mobilization but with drain clamped shut and fixated to head/neck level	Positioning postoperatively in supine position with the upper body elevated 30°. From 6 hours postoperatively onward, patients are allowed to sit up and go on short walks, without clamping the drain shut^14-16^
17	Physiotherapy and ergotherapy	In all cases with focal deficits, cognitive deficits, or gait insecurity	In all cases with focal deficits, cognitive deficits, or gait insecurity
18	Urinary catheters	Early removal or avoidance	Early removal or avoidance
19	Discharge disposition	According to an interdisciplinary decision among treating physicians, physiotherapists, and ergotherapists, patients are either discharged home, to a (temporary) nursing home, or to a rehabilitation clinic	According to an interdisciplinary decision among treating physicians, physiotherapists, and ergotherapists, patients are either discharged home, to a (temporary) nursing home, or to a rehabilitation clinic. In appropriate cases, early transfer to a geriatric ward is recommended
20	Postoperative imaging during hospital stay	Noncontrast computed tomography is recommended before discharge	No imaging up to discharge as long as clinically stable^18^
Postdischarge			
21	Follow-up regimen	Follow-up clinic visit and noncontrast computed tomography 4–8 weeks after surgery	Follow-up clinic visit and noncontrast computed tomography 6 weeks after surgery
22	Removal of sutures/staples	On postoperative day 8 if wounds are nonirritated	On postoperative day 8 if wounds are nonirritated
23	Restart of oral anticoagulants/antiaggregants	Oral anticoagulants or antiaggregants are only started after the 4- to 6-week follow-up visit	Oral anticoagulants or antiaggregants are restarted 1 day after removal of sutures/staples^19-24^

ERAS, enhanced recovery after surgery; I.U., international units; i.v., intravenous; s.c., subcutaneous.

## METHODS

### Overview and Data Collection

**FIGURE 1.**
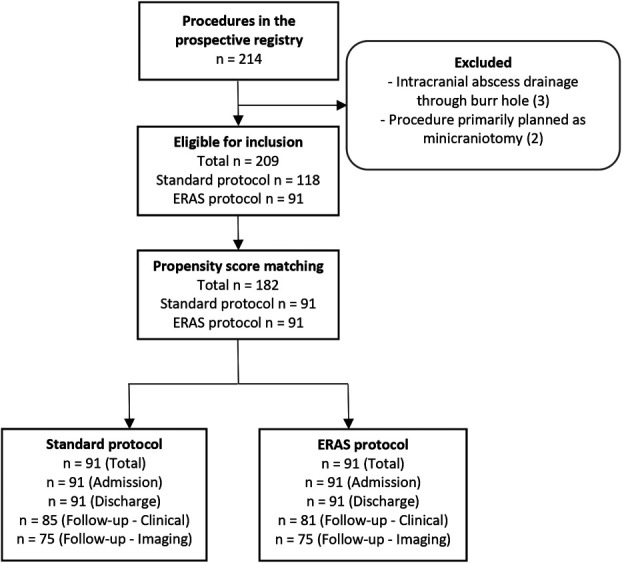
Flowchart demonstrating the flow of procedures collected in the prospective institutional registry up to statistical analysis. ERAS, enhanced recovery after surgery.

Our institutional protocol was changed from the previous “standard protocol” to the novel ERAS protocol and implemented from October 3rd, 2022, onward. To evaluate the real-world safety and effectiveness of the protocol, we analyze all adult patients who have undergone burr hole surgery for subdural chronic hematoma or hygroma at the Department of Neurosurgery of the University Hospital Zurich, from October 3rd, 2022, up to June 2023, as this allowed these patients to complete the 6-week follow-up visit. We excluded procedures done for subdural empyema/abscesses or those primarily planned as craniotomies (Figure 1). We then extracted data for all patients undergoing the same procedure—under the “standard protocol”—during the preceding year, thus from October 2021 up to October 2022. To increase the comparability of the 2 cohorts, propensity score–based matching is carried out. All analyses are performed under the intention-to-treat (ITT) principle. Thus, all eligible patients operated within the active time frame of the “standard” or the novel protocol are analyzed in that group—regardless of whether any or all of the elements were actually implemented for that specific patient. Data extraction is based on our prospective institutional patient registry, which collects demographic, procedural, adverse events, and clinical outcome data. Missing data and imaging or anticoagulant/antiaggregant data are retrospectively added. Ethical approval was obtained for our research registry (KEK-ZH-PB-2017-00093). All patients consented to research use of their data.

### Interventions

#### Protocols

A detailed overview of the “standard” vs the novel enhanced recovery protocol, detailing all differences, is provided in Table 1.^9-26^ Major differences are as follows:

1. Thromboprophylaxis is started 6 hours postoperatively, instead of after 48 hours after drain removal
2. Patients postoperatively rest with the upper body 30° elevated, instead of in flat supine position
3. After 6 hours, patients are allowed to sit up and mobilize, instead of strict bed rest until drain removal
4. When sitting up or mobilizing, the drain does not need to be clamped, instead of it having to be clamped shut before
5. The drainage bag is generally held at heart level, instead of fixated to the bed at neck or shoulder level
6. Oral anticoagulants and antiaggregants are started again 1 day after suture/staple removal (which is done on postoperative day 8), instead of only after the clinical follow-up visit 4–8 weeks after surgery

#### Surgical Technique

The patient is positioned in supine or semilateral position with the upper body and head slightly elevated and turned so that the frontal burr hole corresponds to the highest point of the head. For bilateral procedures, the patient is positioned supine with the head on a half-moon headrest. The Stephanion and parietal tuberosity are identified, and short skin incisions made over each. After dissecting the periosteum, 14-mm burr holes are placed over the Stephanion and the parietal tuberosity. Hemostasis is achieved using bone wax. The dura is incised in a cruciform fashion and coagulated, first frontally and then parietally. Ringer's solution at body temperature is flushed from both burr holes until good communication is achieved between the 2 and until the solution flows back clearly. A Redon drainage is then placed subperiosteally, covering both burr holes. The parietal incision is then closed with inverted galeal sutures and the skin closed with staples. Finally, the remaining subdural space is filled with Ringer's solution until no air remains subdurally, and the frontal incision is closed in the same fashion.

### Outcome Measures

#### Baseline Characteristics

Apart from basic demographic characteristics, we collected American Society of Anesthesiologists scores, emergency vs elective procedures, presence of subdural hematoma vs hygroma, unilateral or bilateral surgery, conversion to craniotomy, previous surgery for cSDH, surgical time in minutes, preoperative epileptic episodes, and preoperative presence of anticoagulants such as vitamin K inhibitors or direct FXa inhibitors, as well as antiaggregants such as acetylsalicylic acid or ADP/P2Y inhibitors.

#### Primary End Points

The primary safety end point (both measured at discharge) was defined as any adverse event as graded by the Clavien–Dindo (CDG) classification,^27^ while the primary effectiveness end point was defined as functional-neurological outcome as graded by the modified Rankin Scale (mRS).

#### Secondary End Points

As secondary clinical outcomes, we collected Glasgow Coma Scale (GCS), Karnofsky Performance Status (KPS), and National Institutes of Health Stroke Scale as well as presence of focal sensorimotor deficits and all-cause mortality up to first follow-up. In cases of mortality, we defined mRS as 6, KPS as 0, GCS as 3, and CDG as V. We also recorded time up to drain removal, time from procedure to discharge, and total length of hospital stay in days. Discharge disposition was also recorded. Finally, in terms of imaging outcomes, we recorded absolute and dynamic evacuation results on noncontrast computed tomography (CT) at first follow-up, once as an absolute classification (complete resorption/residual hematoma ≤2 mm or ≥2 mm/unchanged or increased thickness), and once as a comparison to preoperative imaging (complete resorption or decrease/unchanged thickness/increased thickness). For both measurements, the maximum residual thickness of the hematoma was measured. In cases of bilateral surgery, the thicker residual was counted.

### Statistical Analysis

Categorical variables are reported as numbers (percentages). Distribution of continuous variables is checked using histograms; all are found not to be approximately normally distributed. Continuous data are reported as medians IQRs. Analyses are carried out per procedure—not per patient—and group assignment according to the ITT principle. All eligible patients are pooled, and 2 optimal groups are constructed in a 1:1 ratio using nearest-neighbor propensity score–based matching^28^ for sex, age, emergency surgery, bilateral or unilateral surgery, GCS at admission, presence of hematoma vs hygroma, intake of anticoagulant drugs, and presence of preoperative epileptic events. An overview of prematching data is provided in **Supplemental Digital Content 1, Tables 1-3** (http://links.lww.com/NEU/E104). No imputation was carried out. Intergroup comparisons were then made using Mann-Whitney *U* tests for continuous and ordinal, as well as Pearson's χ^2^ tests for categorical variables. All analyses were performed using version 4.3.1 of R (R Foundation for Statistical Computing; https://www.R-project.org/).^29^ A 2-tailed *P* ≤ .05 was considered statistically significant.

## RESULTS

**FIGURE 2.**
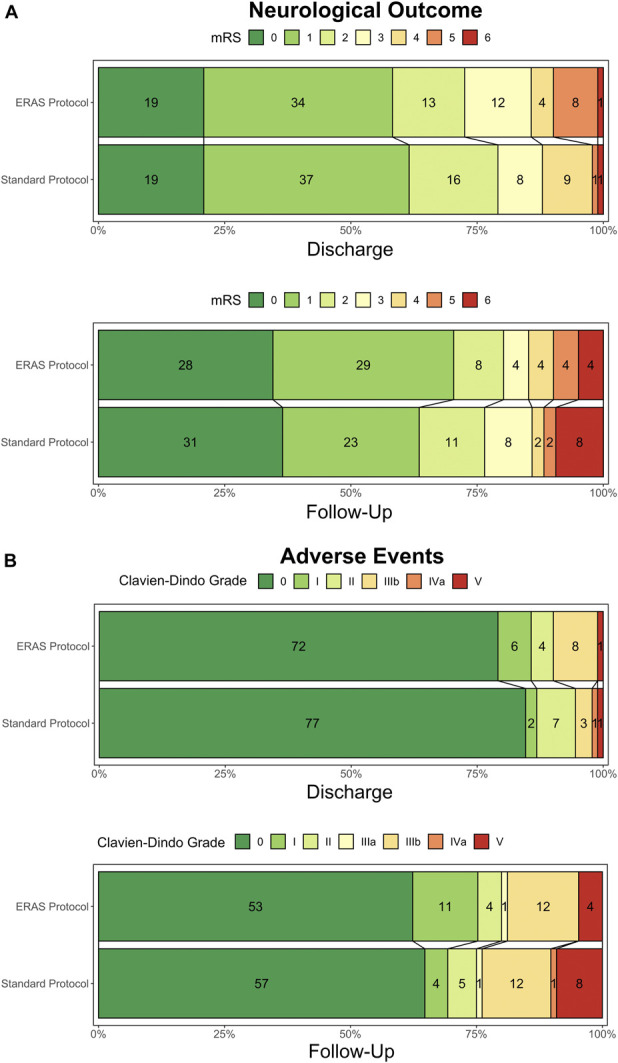
Grotta bar charts demonstrating **A**, the primary effectiveness (functional-neurological outcome as graded by the modified Rankin Scale) and **B**, safety (adverse events as graded by the classification described by Clavien and Dindo) at discharge and at first follow-up. ERAS, enhanced recovery after surgery; mRS, modified Rankin Scale.

Data on a total of 209 eligible procedures were available (Figure 1), of which 118 in the standard and 91 in the enhanced recovery protocol group. After matching, 91 procedures were available per group. Table 2 provides an overview of baseline characteristics, without evidence of differential distribution among the 2 groups, apart from median surgical time, which was significantly longer in the standard protocol vs the enhanced recovery group (57.0 [45.0; 70.0] vs 45.0 [36.0; 60.0] minutes, *P* < .001). Overall, 3.3% of procedures were carried out under local anesthesia, without significant intergroup difference (5.5% vs 1.1%, *P* = .213). Data on clinical first follow-up were lost for 16 procedures (8.8%), and the median length of follow-up in weeks was 6 [5; 8]. Primary end point results are illustrated in Figure 2.

**TABLE 2.** Summary of Baseline Demographic, Procedural, Discharge, and Follow-up Characteristics

Parameter	Overall (N = 182)	Standard protocol (N = 91)	ERAS protocol (N = 91)	*P*
Age [y], (median [IQR])	77.00 [67.00, 84.00]	77.00 [66.00, 84.00]	76.00 [67.00, 82.00]	.358
Male gender, n (%)	133 (73.1)	66 (72.5)	67 (73.6)	1.000
ASA score, n (%)				.729
II	19 (10.4)	10 (11.0)	9 (9.9)	
III	130 (71.4)	63 (69.2)	67 (73.6)	
IV	32 (17.6)	17 (18.7)	15 (16.5)	
V	1 (0.5)	1 (1.1)	0 (0.0)	
Emergency procedure, n (%)	113 (62.1)	54 (59.3)	59 (64.8)	.541
cSDH (vs hygroma), n (%)	168 (92.3)	88 (96.7)	80 (87.9)	.052
Bilateral surgery, n (%)	52 (28.6)	28 (30.8)	24 (26.4)	.623
Conversion to craniotomy, n (%)	1 (0.5)	1 (1.1)	0 (0.0)	1.000
Previous surgery for cSDH, n (%)	17 (9.3)	7 (7.7)	10 (11.0)	.610
Anticoagulation, n (%)	57 (31.3)	22 (24.2)	35 (38.5)	.055
Antiaggregation, n (%)	45 (24.7)	22 (24.2)	23 (25.3)	1.000
Surgical time [min], (median [IQR])	51.50 [40.00, 65.00]	57.00 [45.00, 70.00]	45.00 [36.00, 60.00]	<.001^a^
Procedures under local anesthesia, n (%)	6 (3.3)	5 (5.5)	1 (1.1)	.213
Drain removed on POD [d], (median [IQR])	2.00 [2.00, 2.00]	2.00 [2.00, 2.00]	2.00 [1.25, 2.00]	.058
Discharge characteristics				
Time from procedure to discharge [d], (median [IQR])	4.00 [3.00, 6.00]	4.00 [3.00, 6.00]	4.00 [3.00, 6.00]	.201
Total hospital length of stay [d], (median [IQR])	6.00 [5.00, 8.75]	6.00 [5.00, 9.00]	5.00 [4.00, 8.00]	.113
Discharge disposition, n (%)				.192
Home	105 (57.7)	58 (63.7)	47 (51.6)	
Rehabilitation clinic	22 (12.1)	8 (8.8)	14 (15.4)	
Other acute care hospital	41 (22.5)	16 (17.6)	25 (27.5)	
Care facility/nursing home	12 (6.6)	8 (8.8)	4 (4.4)	
In-hospital death	2 (1.1)	1 (1.1)	1 (1.1)	
Follow-up characteristics				
Length of first FU [wk], (median [IQR])	6.00 [5.00, 8.00]	6.00 [5.00, 8.00]	6.00 [6.00, 8.00]	.952
Lost to FU, n (%)	16 (8.8)	6 (6.6)	10 (11.0)	.432
All-cause mortality up to first FU, n (%)	12 (6.6)	8 (8.8)	4 (4.4)	.289
Missingness, n (%)	1 (0.5)	1 (1.1)	0 (0.0)	

ASA, American Society of Anesthesiologists; cSDH, chronic subdural hematoma; ERAS, enhanced recovery after surgery; FU, follow-up; POD, postoperative day.

a *P* ≤ .05.

### Primary End Points

#### Effectiveness—Functional Neurological Outcome

At discharge, there was no significant difference in the mRS among the standard and ERAS groups (1 [1; 2] vs 1 [1; 3], *P* = .552, Table 3). Similarly, there was no difference in mRS at first follow-up (1 [0; 2] vs 1 [0; 2], *P* = .737).

**TABLE 3.** Summary of Clinical Outcome Data at Admission, Discharge, and First Follow-up

Parameter	Overall (N = 182)	Standard protocol (N = 91)	ERAS protocol (N = 91)	*P*
Admission				
GCS, (median [IQR])	15.00 [14.00, 15.00]	15.00 [14.00, 15.00]	15.00 [14.00, 15.00]	.919
mRS, (median [IQR])	2.00 [1.00, 3.00]	2.00 [1.00, 3.00]	2.00 [1.00, 3.00]	.825
KPS, (median [IQR])	80.00 [60.00, 80.00]	80.00 [60.00, 80.00]	80.00 [60.00, 80.00]	.968
NIHSS, (median [IQR])	0.00 [0.00, 2.00]	0.00 [0.00, 2.00]	0.00 [0.00, 1.25]	.329
Missingness, n (%)	73 (40.1)	42 (46.2)	31 (34.1)	
Focal sensorimotor deficit, n (%)	97 (53.3)	50 (54.9)	47 (51.6)	.766
Preop. epileptic episodes, n (%)	38 (20.9)	21 (23.1)	17 (18.7)	.584
Discharge				
GCS, (median [IQR])	15.00 [15.00, 15.00]	15.00 [15.00, 15.00]	15.00 [15.00, 15.00]	.475
mRS, (median [IQR])	1.00 [1.00, 2.00]	1.00 [1.00, 2.00]	1.00 [1.00, 3.00]	.552
KPS, (median [IQR])	90.00 [80.00, 90.00]	90.00 [80.00, 90.00]	90.00 [75.00, 90.00]	.347
NIHSS, (median [IQR])	0.00 [0.00, 0.00]	0.00 [0.00, 0.00]	0.00 [0.00, 0.00]	.649
Missingness, n (%)	51 (28.0)	33 (36.3)	18 (19.8)	
Focal sensorimotor deficit, n (%)	50 (27.5)	28 (30.8)	22 (24.2)	.406
First follow-up				
GCS, (median [IQR])	15.00 [15.00, 15.00]	15.00 [15.00, 15.00]	15.00 [15.00, 15.00]	.544
Missingness, n (%)	16 (8.8)	6 (6.6)	10 (11.0)	
mRS, (median [IQR])	1.00 [0.00, 2.00]	1.00 [0.00, 2.00]	1.00 [0.00, 2.00]	.737
Missingness, n (%)	16 (8.8)	6 (6.6)	10 (11.0)	
KPS, (median [IQR])	90.00 [80.00, 100.00]	90.00 [80.00, 100.00]	90.00 [80.00, 100.00]	.789
Missingness, n (%)	16 (8.8)	6 (6.6)	10 (11.0)	
NIHSS, (median [IQR])	0.00 [0.00, 0.00]	0.00 [0.00, 0.00]	0.00 [0.00, 0.00]	.514
Missingness, n (%)	65 (35.7)	34 (37.4)	31 (34.1)	
Focal sensorimotor deficit, n (%)	46 (25.3)	26 (28.6)	20 (22.0)	.220
Missingness, n (%)	16 (8.8)	5 (5.5)	11 (12.1)	

ERAS, enhanced recovery after surgery; GCS, Glasgow Coma Scale; mRS, modified Rankin Scale; KPS, Karnofsky Performance Status; NIHSS, National Institutes of Health Stroke Scale.

#### Safety—Adverse Events

At discharge, there was no significant difference in CDG classifications of adverse events (*P* = .282, Table 4) or in the occurrence of any adverse events (15.4% vs 20.9%, *P* = .442). Similar results were observed at follow-up for CDG classifications (*P* = .444) or occurrence of any adverse events (34.1% vs 35.2%, *P* = .560).

**TABLE 4.** Summary of Adverse Events (at Discharge and at First Follow-up) as Well as Imaging Outcomes (at First Follow-up) on Computed Tomography

Parameter	Overall (N = 182)	Standard protocol (N = 91)	ERAS protocol (N = 91)	*P*
Adverse events at discharge				
Any adverse event, n (%)	33 (18.1)	14 (15.4)	19 (20.9)	.442
Clavien–Dindo classification, n (%)				.282
0	149 (81.9)	77 (84.6)	72 (79.1)	
I	8 (4.4)	2 (2.2)	6 (6.6)	
II	11 (6.0)	7 (7.7)	4 (4.4)	
IIIb	11 (6.0)	3 (3.3)	8 (8.8)	
IVa	1 (0.5)	1 (1.1)	0 (0.0)	
V	2 (1.1)	1 (1.1)	1 (1.1)	
Adverse events at first follow-up				
Any adverse event, n (%)	63 (34.6)	31 (34.1)	32 (35.2)	.560
Missingness, n (%)	9 (4.9)	3 (3.3)	6 (6.6)	
Clavien–Dindo classification, n (%)				.444
0	110 (60.4)	57 (62.6)	53 (58.2)	
I	15 (8.2)	4 (4.4)	11 (12.1)	
II	9 (4.9)	5 (5.5)	4 (4.4)	
IIIa	2 (1.1)	1 (1.1)	1 (1.1)	
IIIb	24 (13.2)	12 (13.2)	12 (13.2)	
IVa	1 (0.5)	1 (1.1)	0 (0.0)	
V	12 (6.6)	8 (8.8)	4 (4.4)	
Missingness, n (%)	9 (4.9)	3 (3.3)	6 (6.6)	
Outcomes on imaging at first follow-up				
Absolute max. hematoma thickness, n (%)				.471
Complete resorption	24 (13.2)	16 (17.6)	8 (8.8)	
Residual hematoma ≤2 mm	11 (6.0)	6 (6.6)	5 (5.5)	
Residual hematoma >2 mm	103 (56.6)	47 (51.6)	56 (61.5)	
Unchanged or increased hematoma	12 (6.6)	6 (6.6)	6 (6.6)	
Missingness, n (%)	32 (17.6)	16 (17.6)	16 (17.6)	
Dynamic max. hematoma thickness^a^ (%)				.952
Decreased or complete resorption	138 (75.8)	69 (75.8)	69 (75.8)	
Unchanged	7 (3.8)	3 (3.3)	4 (4.4)	
Increased	5 (2.7)	3 (3.3)	2 (2.2)	
Missingness, n (%)	32 (17.6)	16 (17.6)	16 (17.6)	

ERAS, enhanced recovery after surgery.

a Compared with baseline imaging.

### Secondary End Points

There were no significant differences in time to drain removal (2.00 [2.00; 2.00] vs 2.00 [1.25; 2.00] days, *P* = .058), time from procedure to discharge (4.0 [3.0; 6.0] vs 4.0 [3.0; 6.0] days, *P* = .201), or total hospital length of stay (6.0 [5.0; 9.0] vs 5.0 [4.0; 8.0] days, *P* = .113). All-cause mortality up to first follow-up was similar in both groups (8.8% vs 4.4%, *P* = .289), as was discharge disposition (*P* = .192). In terms of other clinical outcomes, there were no significant differences in GCS, KPS, National Institutes of Health Stroke Scale, or focal sensorimotor deficits, while imaging outcomes were similar too (all *P* > .05).

## DISCUSSION

In this matched comparison of an enhanced recovery protocol for burr hole evacuation of cSDH vs its standard predecessor based on a prospective single-center registry, there was no evidence for superiority or inferiority of the novel protocol in terms of safety or effectiveness.

Strategies to improve recovery after surgery have not only demonstrated their benefits in various specialties, including in spinal and—to a lesser extent—also in cranial neurosurgery, but they may be of particular importance in those patient populations who show high levels of frailty and multimorbidity, and thus are more prone to hospital-acquired adverse events.^5,8^ Patients with cSDH fit this description relatively well, and there is very limited evidence on such strategies in this specific group of patients.^1^ One crucial characteristic of the ERAS concept is that each intervention that is implemented as an element within a protocol must be at least somewhat evidence-based. Although there are as of yet no published ERAS protocols for burr hole evacuation of cSDH, some evidence on specific interventions that could be combined into a multimodal protocol does exist.

In our protocol, we start thromboprophylaxis using heparin 6 hours postoperatively, whereas we would only do so after 48 hours in our previous protocol. This decision was based on the retrospective single-center study by Licci et al,^16^ which demonstrated no difference in the recurrence rate after evacuation of cSDH whether thromboprophylaxis was started before or after 48 hours. Their study, however, did find a correlation of increased doses of thromboprophylaxis leading to increased recurrence rates. Our data corroborate these findings, demonstrating no difference in imaging or clinical outcomes regardless of administration time point. However, there was also no clear benefit in starting thromboprophylaxis earlier, as complication rates—including thromboses—were also unchanged. Nonetheless, it appears safe to start a heparin drip 6 hours postoperatively.

The protocol also allowed a 30° upper-body elevation as opposed to flat bed rest, and allowed early mobilization after 6 hours as opposed to strict bed rest until drain removal. A multicenter prospective study by Brennan et al^13^ demonstrated that postoperative bed was an independent predictor of unfavorable functional outcome, while Kurabe et al^14^ show that upright positioning and mobilization on the same day as surgery led to decreased complications while maintaining the same recurrence rate in a retrospective single-center study. The randomized controlled trial (RCT) by Nakajima et al^30^ demonstrated no effect of postoperative posture on recurrence, while the RCT by Abouzari et al^31^ saw a slight increase in recurrence rates with upright vs supine positioning. In addition, a meta-analysis of 4 studies by Zhu et al^15^ found that postoperative bed header position (upright or flat) had no effect on recurrence rates. These previous studies fit well with our observations and in summary indicate that it is likely safe to allow patients to sit in an upright position and to mobilize patients on the day of surgery.

Furthermore, the novel protocol specified that oral anticoagulant and antiaggregant drugs ought to be started 1 day after suture/staple removal—thus usually on the ninth postoperative day and after early wound complications that might require revision are ruled out—as opposed to only after a 4- to 8-week clinical visit with noncontrast CT imaging. Multiple studies have assessed the optimal management of these drugs in the perioperative period.^18-23^ For example, Fornebo et al^19^ carried out a retrospective study of 763 patients and found that recurrence rates were not significantly different between early vs late (≤30 vs >30 days) resumption of anticoagulants or antiaggregants, but that the late resumption group did have significantly more thromboembolic complications. Retrospective studies by Amano et al^18^ and Guha et al^32^ demonstrate that—in this population—severe thromboembolic events occur usually within the first month after surgery, especially in those patients who were on anticoagulant drugs before cSDH evacuation. The latter of the 2 also provides some evidence that it is safe to restart anticoagulants and antiaggregants at 3 days postoperatively.^32^ A meta-analysis by Phan et al^21^ demonstrated no difference in hemorrhagic complications when resuming antithrombotic agents in <2 weeks vs >1 month, while earlier resumption did lead to significantly lower thromboembolic complications.

These data show that implementing our protocol—at least in its earliest stages of adoption—did not affect adverse events or any type of outcome, neither positively nor negatively. Of course, it has to be considered that “soft” outcomes such as patient comfort and satisfaction are not considered here, or that adherence to the protocol elements in truth was perhaps lower than expected in these first months of adoption, as adherence usually increases over time.^7,33^ It is also noteworthy that length of stay and discharge disposition—2 further important outcomes concerning ERAS—were unchanged. One potential reason that impact was low here could be inherent to the Swiss health system. The threshold for referral to a rehabilitation clinic is low and postoperative waiting times for a bed in a rehabilitation clinic are long. Thus, a large proportion of patients with neurocognitive or focal deficits are either sent to rehabilitation after a prolonged hospital stay, or are transferred to regional hospitals, to wait for a rehabilitation bed there.

It still is difficult to find appropriate, high-level evidence for each single element of clinical care, such as how many days exactly one should wait until resuming antithrombotic drugs, or what height exactly the drainage bag should be fixed to. Likely, it will also remain impossible to ever generate such high-level evidence for any such decision, using RCTs or large, multicenter, prospective studies. This is one of the reasons why much of ERAS research—and inherently so, surgical science in general—must also rely to a certain extent on pragmatic studies reporting the real-world experience of a surgical team with a protocol that is as clearly defined and as standardized as it is possible in actual clinical practice. Such studies aiming to evaluate the *effectiveness*^34^ (thus, the real-world performance of an intervention in a broad patient group) can still be crucial in guiding surgical management, which cannot always be represented in studies aiming to evaluate *efficacy* (thus, the performance of an intervention under research circumstances, such as in a RCT with narrowly defined inclusion and exclusion criteria).

Although our data provide some evidence on a novel enhanced recovery protocol for patients undergoing evacuation of cSDH, further prospective, multicenter studies with larger samples are required to validate the safety and effectiveness of our protocol with a higher level of evidence. In addition, RCTs could provide a high level of evidence regarding its efficacy and are probably necessary before such protocols can be more widely adopted clinically. It will also be crucial to validate novel elements that can then be added to protocols, then finally also comparing different protocols with each other.

### Limitations

The most important limitation of this study is the lack of an analysis of protocol adherence. It is known that the most important factor determining the impact of ERAS protocols is the percentage of adherence to the single ERAS elements.^33^ It is likely that not all elements have been strictly adhered to in each patient, and it is possible that subsets of patients with higher adherence might demonstrate improved outcomes. Furthermore, we used propensity score–based matching to increase the comparability of the 2 study groups. However, matching can only account for confounders that are collected and indeed matched for, in contrast to, eg, a randomized study design that equally distributes all confounders. In addition, it was necessary to supplement some data retrospectively, such as imaging results and antiaggregant/anticoagulant drug intake. Still, the majority of outcomes—importantly including our primary end points—were prospectively collected. It is known that especially regarding complications, prospective registries tend to result in significantly higher complication rates than retrospective analysis of adverse events. Our study is also not powered to demonstrate numerically small but potentially clinically important differences, which would require a far larger number of included patients.

## CONCLUSION

In a matched cohort study comparing perioperative standard of care with a novel enhanced recovery protocol focusing on evidence-based drainage, mobilization, and thromboprophylaxis regimens as well as changes to the standardized reuptake of oral anticoagulants and antiaggregants, no differences in safety or effectiveness were observed after burr hole evacuation of cSDH.

## Supplementary Material

SUPPLEMENTARY MATERIAL

## SUPPLEMENTAL DIGITAL CONTENT

**Supplemental Digital Content 1. Table 1**. Summary of baseline demographic, procedural, discharge, and follow-up characteristics, before propensity score–based matching (entire cohort). ERAS, enhanced recovery after surgery; IQR, interquartile range; ASA, American Society of Anesthesiologists; cSDH, chronic subdural hematoma; POD, postoperative day; FU, follow-up; **P* ≤ .05. **Table 2.** Summary of clinical outcome data at admission, discharge, and first follow-up, before propensity score–based matching (entire cohort). ERAS, enhanced recovery after surgery; GCS, Glasgow Coma Scale; IQR, interquartile range; mRS, modified Rankin Scale; KPS, Karnofsky Performance Status; NIHSS, National Institutes of Health Stroke Scale. **Table 3.** Summary of adverse events (at discharge and at first follow-up) as well as imaging outcomes (at first follow-up) on computed tomography, before propensity score–based matching (entire cohort). ERAS, enhanced recovery after surgery; * Compared with baseline imaging.
