# Supplementary material for: Safety and Effectiveness of an Enhanced Recovery Protocol in Patients Undergoing Burr Hole Evacuation for Chronic Subdural Hematoma
Source: Neurosurgery. 2024 Feb 7;95(1):146–57. doi: 10.1227/neu.0000000000002849 (PMC11155564; doi:10.1227/neu.0000000000002849)
Supplement: SUPPLEMENTARY MATERIAL [file neu-95-146-s001.docx]

**Supplemental Digital Content 1. Table 1.** Summary of baseline demographic, procedural, discharge, and follow-up characteristics, before propensity score-based matching (entire cohort).

|  | **Overall**  **(N = 209)** | **Standard Protocol**  **(N = 118)** | **ERAS Protocol**  **(N = 91)** |
| --- | --- | --- | --- |
| **Age [yrs.], (median [IQR])** | 78.00 [67.00, 84.00] | 79.50 [67.50, 85.00] | 76.00 [67.00, 82.00] |
| **Male gender, n (%)** | 155 (74.2) | 88 (74.6) | 67 (73.6) |
| **ASA score, n (%)** |  |  |  |
| II | 20 (9.6) | 11 (9.3) | 9 (9.9) |
| III | 150 (71.8) | 83 (70.3) | 67 (73.6) |
| IV | 37 (17.7) | 22 (18.6) | 15 (16.5) |
| V | 2 (1.0) | 2 (1.7) | 0 (0.0) |
| **Emergency procedure, n (%)** | 134 (64.1) | 75 (63.6) | 59 (64.8) |
| **cSDH (vs. Hygroma), n (%)** | 195 (93.3) | 115 (97.5) | 80 (87.9) |
| **Bilateral surgery, n (%)** | 63 (30.1) | 39 (33.1) | 24 (26.4) |
| **Conversion to craniotomy, n (%)** | 1 (0.5) | 1 (0.8) | 0 (0.0) |
| **Procedure under local anesthesia, n (%)** | 6 (2.9) | 5 (4.2) | 1 (1.1) |
| **Prior surgery for cSDH, n (%)** | 23 (11.0) | 13 (11.0) | 10 (11.0) |
| **Anticoagulation, n (%)** | 57 (27.3) | 22 (18.6) | 35 (38.5) |
| **Antiaggregation, n (%)** | 54 (25.8) | 31 (26.3) | 23 (25.3) |
| **Surgical time [mins.], (median [IQR])** | 55.00 [41.00, 66.00] | 60.00 [45.00, 71.50] | 45.00 [36.00, 60.00] |
| **Drain removed on POD [d.], (median [IQR])** | 2.00 [2.00, 2.00] | 2.00 [2.00, 2.00] | 2.00 [1.25, 2.00] |
| **Discharge Characteristics** |  |  |  |
| **Time from procedure to discharge [d.], (median [IQR])** | 4.00 [3.00, 6.00] | 4.00 [3.00, 6.00] | 4.00 [3.00, 6.00] |
| **Total hospital length of stay [d.], (median [IQR])** | 6.00 [5.00, 9.00] | 6.00 [5.00, 10.00] | 5.00 [4.00, 8.00] |
| **Discharge disposition, n (%)** |  |  |  |
| Home | 114 (54.5) | 67 (56.8) | 47 (51.6) |
| Rehabilitation Clinic | 30 (14.4) | 16 (13.6) | 14 (15.4) |
| Other acute care hospital | 47 (22.5) | 22 (18.6) | 25 (27.5) |
| Care facility / Nursing home | 16 (7.7) | 12 (10.2) | 4 (4.4) |
| In-hospital death | 2 (1.0) | 1 (0.8) | 1 (1.1) |
| **Follow-Up (FU) Characteristics** |  |  |  |
| **Length of first FU [wks.], (median [IQR])** | 6.00 [5.00, 8.00] | 6.00 [5.00, 8.00] | 6.00 [6.00, 8.00] |
| **Lost to FU, n (%)** | 17 (8.1) | 7 (5.9) | 10 (11.0) |
| **All-cause mortality up to first FU, n (%)** | 15 (7.2) | 11 (9.3) | 4 (4.4) |
| *Missingness, n (%)* | 1 (0.5) | 1 (0.8) | 0 (0.0) |

*ERAS = enhanced recovery after surgery; IQR = interquartile range; ASA = American Society of Anesthesiologists; cSDH = chronic subdural hematoma; POD = postoperative day; FU = follow-up;*

** p ≤ 0.05*

**Supplemental Digital Content 1.Table 2** Summary of clinical outcome data at admission, discharge, and first follow-up, before propensity score-based matching (entire cohort).

|  | **Overall**  **(N = 209)** | **Standard Protocol**  **(N = 118)** | **ERAS Protocol**  **(N = 91)** |
| --- | --- | --- | --- |
| **Admission** |  |  |  |
| **GCS, (median [IQR])** | 15.00 [15.00, 15.00] | 15.00 [15.00, 15.00] | 15.00 [15.00, 15.00] |
| **mRS, (median [IQR])** | 1.00 [1.00, 3.00] | 1.00 [1.00, 2.75] | 1.00 [1.00, 3.00] |
| **KPS, (median [IQR])** | 90.00 [70.00, 90.00] | 90.00 [72.50, 90.00] | 90.00 [75.00, 90.00] |
| **NIHSS, (median [IQR])** | 0.00 [0.00, 0.00] | 0.00 [0.00, 0.00] | 0.00 [0.00, 0.00] |
| **Focal sensorimotor deficit, n (%)** | 63 (30.1) | 41 (34.7) | 22 (24.2) |
| **Preop. epileptic episodes, n (%)** | 15.00 [15.00, 15.00] | 15.00 [15.00, 15.00] | 15.00 [15.00, 15.00] |
| **Discharge** |  |  |  |
| **GCS, (median [IQR])** | 15.00 [15.00, 15.00] | 15.00 [15.00, 15.00] | 15.00 [15.00, 15.00] |
| **mRS, (median [IQR])** | 1.00 [1.00, 3.00] | 1.00 [1.00, 2.75] | 1.00 [1.00, 3.00] |
| **KPS, (median [IQR])** | 90.00 [70.00, 90.00] | 90.00 [72.50, 90.00] | 90.00 [75.00, 90.00] |
| **NIHSS, (median [IQR])** | 0.00 [0.00, 0.00] | 0.00 [0.00, 0.00] | 0.00 [0.00, 0.00] |
| **Focal sensorimotor deficit, n (%)** | 63 (30.1) | 41 (34.7) | 22 (24.2) |
| **First Follow-Up** |  |  |  |
| **GCS, (median [IQR])** | 15.00 [15.00, 15.00] | 15.00 [15.00, 15.00] | 15.00 [15.00, 15.00] |
| **mRS, (median [IQR])** | 1.00 [0.00, 2.00] | 1.00 [0.00, 2.75] | 1.00 [0.00, 2.00] |
| **KPS, (median [IQR])** | 90.00 [80.00, 100.00] | 90.00 [70.00, 100.00] | 90.00 [80.00, 100.00] |
| **NIHSS, (median [IQR])** | 0.00 [0.00, 0.00] | 0.00 [0.00, 0.00] | 0.00 [0.00, 0.00] |
| **Focal sensorimotor deficit, n (%)** | 61 (29.2) | 41 (34.7) | 20 (22.0) |
| *Missingness, n (%)* | 19 (9.1) | 8 (6.8) | 11 (12.1) |

*ERAS = enhanced recovery after surgery; GCS = Glasgow Coma Scale; IQR = interquartile range; mRS = modified Rankin Scale; KPS = Karnofsky Performance Status; NIHSS = National Institutes of Health Stroke Scale;*

**Supplemental Digital Content 1. Table 3** Summary of adverse events (at discharge and at first follow-up) as well as imaging outcomes (at first follow-up) on computed tomography, before propensity score-based matching (entire cohort).

|  | **Overall**  **(N = 209)** | **Standard Protocol**  **(N = 118)** | **ERAS Protocol**  **(N = 91)** |
| --- | --- | --- | --- |
| **Adverse Events at Discharge** |  |  |  |
| **Any adverse event, n (%)** | 43 (20.6) | 24 (20.3) | 19 (20.9) |
| **Clavien-Dindo classification, n (%)** |  |  |  |
| 0 | 166 (79.4) | 94 (79.7) | 72 (79.1) |
| I | 13 (6.2) | 7 (5.9) | 6 (6.6) |
| II | 13 (6.2) | 9 (7.6) | 4 (4.4) |
| IIIb | 13 (6.2) | 5 (4.2) | 8 (8.8) |
| IVa | 1 (0.5) | 1 (0.8) | 0 (0.0) |
| V | 3 (1.4) | 2 (1.7) | 1 (1.1) |
| **Adverse Events at First Follow-Up** |  |  |  |
| **Any adverse event, n (%)** | 77 (36.8) | 45 (38.1) | 32 (35.2) |
| *Missingness, n (%)* | 10 (4.8) | 4 (3.4) | 6 (6.6) |
| **Clavien-Dindo classification, n (%)** |  |  |  |
| 0 | 77 (36.8) | 45 (38.1) | 32 (35.2) |
| I | 10 (4.8) | 4 (3.4) | 6 (6.6) |
| II | 77 (36.8) | 45 (38.1) | 32 (35.2) |
| IIIa | 10 (4.8) | 4 (3.4) | 6 (6.6) |
| IIIb | 77 (36.8) | 45 (38.1) | 32 (35.2) |
| IVa | 10 (4.8) | 4 (3.4) | 6 (6.6) |
| V | 77 (36.8) | 45 (38.1) | 32 (35.2) |
| *Missingness, n (%)* | 10 (4.8) | 4 (3.4) | 6 (6.6) |
| **Outcomes on Imaging at First Follow-Up** |  |  |  |
| **Absolute max. hematoma thickness, n (%)** |  |  |  |
| Complete resorption | 26 (12.4) | 18 (15.3) | 8 (8.8) |
| Residual hematoma ≤2 mm | 11 (5.3) | 6 (5.1) | 5 (5.5) |
| Residual hematoma >2 mm | 122 (58.4) | 66 (55.9) | 56 (61.5) |
| Unchanged or increased hematoma | 15 (7.2) | 9 (7.6) | 6 (6.6) |
| *Missingness, n (%)* | 35 (16.7) | 19 (16.1) | 16 (17.6) |
| **Dynamic max. hematoma thickness* (%)** |  |  |  |
| Decreased or complete resorption | 159 (76.1) | 90 (76.3) | 69 (75.8) |
| Unchanged | 9 (4.3) | 5 (4.2) | 4 (4.4) |
| Increased | 6 (2.9) | 4 (3.4) | 2 (2.2) |
| *Missingness, n (%)* | 35 (16.7) | 19 (16.1) | 16 (17.6) |

*ERAS = enhanced recovery after surgery;*

** Compared to baseline imaging*
